# Prediction of Short and Long Survival after Surgery for Breast Cancer Brain Metastases

**DOI:** 10.3390/cancers14061437

**Published:** 2022-03-10

**Authors:** Anna Michel, Marvin Darkwah Oppong, Laurèl Rauschenbach, Thiemo Florin Dinger, Lennart Barthel, Daniela Pierscianek, Karsten H. Wrede, Jörg Hense, Christoph Pöttgen, Andreas Junker, Teresa Schmidt, Antonella Iannaccone, Rainer Kimmig, Ulrich Sure, Ramazan Jabbarli

**Affiliations:** 1 Department of Neurosurgery and Spine Surgery, University Hospital Essen, 45147 Essen, Germany; marvin.darkwahoppong@uk-essen.de (M.D.O.); laurel.rauschenbach@uk-essen.de (L.R.); thiemo-florin.dinger@uk-essen.de (T.F.D.); lennart.barthel@uk-essen.de (L.B.); daniela.pierscianek@uk-essen.de (D.P.); karsten.wrede@uk-essen.de (K.H.W.); ulrich.sure@uk-essen.de (U.S.); ramazan.jabbarli@uk-essen.de (R.J.); 2 Department of Medical Oncology, University Hospital Essen, 45147 Essen, Germany; joerg.hense@uk-essen.de; 3 Department of Radiotherapy, University Hospital Essen, 45147 Essen, Germany; christoph.poettgen@uk-essen.de; 4 Department of Neuropathology, University Hospital Essen, 45147 Essen, Germany; andreas.junker@uk-essen.de; 5 Department of Neurooncology, University Hospital Essen, 45147 Essen, Germany; teresa.schmidt@uk-essen.de; 6 Department of Obstetrics and Gynecology, University Hospital Essen, 45147 Essen, Germany; antonella.iannaccone@uk-essen.de (A.I.); rainer.kimmig@uk-essen.de (R.K.)

**Keywords:** brain metastasis, breast cancer, score, HER2

## Abstract

**Simple Summary:**

The aim of the present retrospective study was to develop a new scoring system for prognosis of patients undergoing surgery for breast cancer brain metastasis. Our institutional cohort (*n* = 95) was analyzed with regard to independent predictors of short (<6 months) and long (≥3 years) survival. Breast-preserving surgery, presence of multiple brain metastases, and age ≥ 65 years at brain cancer diagnosis were associated with short survival. In turn, positive HER2 receptor status in brain metastasis, time interval ≥ 3 years between breast cancer and brain metastasis diagnosis and KPS ≥ 90% were the long survival predictors. The scores based on the above-mentioned independent predictors showed good diagnostic accuracy for the prediction of short (AUC = 0.773) and long (AUC = 0.775) survival. After external validation, the presented scores might become useful tools to support the interdisciplinary decision for the selection of treatment strategy in individuals with breast cancer brain metastases.

**Abstract:**

Background: Brain metastases requiring surgical treatment determine the prognosis of patients with breast cancer. We aimed to develop the scores for the prediction of short (<6 months) and long (≥3 years) survival after BCBM surgery. Methods: Female patients with BCBM surgery between 2008 and 2019 were included. The new scores were constructed upon independent predictors for short and long postoperative survival. Results: In the final cohort (*n* = 95), 18 (18.9%) and 22 (23.2%) patients experienced short and long postoperative survival, respectively. Breast-preserving surgery, presence of multiple brain metastases and age ≥ 65 years at breast cancer diagnosis were identified as independent predictors of short postoperative survival. In turn, positive HER2 receptor status in brain metastases, time interval ≥ 3 years between breast cancer and brain metastases diagnosis and KPS ≥ 90% independently predicted long survival. The appropriate short and long survival scores showed higher diagnostic accuracy for the prediction of short (AUC = 0.773) and long (AUC = 0.775) survival than the breast Graded Prognostic Assessment score (AUC = 0.498/0.615). A cumulative survival score (total score) showed significant association with overall survival (*p* = 0.001). Conclusion: We identified predictors independently impacting the prognosis after BCBM surgery. After external validation, the presented scores might become useful tools for the selection of proper candidates for BCBM surgery.

## 1. Introduction

Breast cancer [1] is one of the most common cancer entities in women with high impact of interest and an incidence of 11 to 24% [2,3,4,5]. Therapy concepts of primary breast cancer and its distant metastases, including the brain metastases [6], consist of the local surgical and (neo-)adjuvant treatments (conventional chemotherapy, endocrine therapy, and radiation), as well as targeted therapy (anti-human epidermal growth factor receptor 2 (HER2)), which improve the patients’ survival and the chances for curability [4,7,8,9,10,11,12,13,14,15]. The receptor status is crucial for therapy concepts and development of distant metastases, making the receptor status an important prognostic factor [4,7,11]. Triple negative breast cancer have the poorest prognosis and, in particular, breast cancer patients with HER2+ or triple negative receptor status are prone to brain metastases [16,17,18]. Breast cancer subtypes also impact the risk of brain metastases development [16,19,20,21,22,23,24]. Depending on the presence of risk factors, 15–50% of breast cancer patients develop brain metastases during the disease course.

In general, breast cancer patients with brain metastases are characterized by a poor prognosis. The median postoperative overall survival in BCBM varies between 7.2 to up to 37.7 months [25,26,27,28]. In BCBM cases selected for microsurgical resection, postoperative treatment should include radiotherapy and systemic therapy, which might be enhanced according to the receptor status of breast cancer [20,22,28,29].

The prognosis of patients with BCBM depends on several factors. Previous studies identified an older age, extracranial metastases, number of brain metastases, lower Karnofsky Performance Status (KPS) scale, and triple-negative breast cancer as major survival predictors [16,17,26,27,30,31,32,33,34,35]. Although not specifically dedicated for BCBM patients selected for microsurgery, the breast Graded Prognostic Assessment Score (GPA) for BCBM allows a cumulative estimation of survival using the following major predictors: breast cancer subtype, KPS, and patient’s age [26,36]. The subsequently presented modified breast-GPA included the number of brain metastases and a modified cutoff for age [37,38,39,40]. Finally, in a recent modification of the GPA-score, the updated breast-GPA score [41], presence of extracranial metastases was also considered. Although the scores can be easily calculated and therefore are clinically practicable, but their diagnostic accuracy for the prediction of postoperative survival in BCBM patients remains unclear.

The aim of the present study was to identify independent prognostic factors for BCBM patients selected for tumor resection, with subsequent construction of the risk scores for short (SS) as and long survival (LS).

## 2. Material and Methods

This study was performed in accordance with the Declaration of Helsinki and approved by the local ethics committee of the University Hospital Essen (local registration number: 17-7855-BO).

### 2.1. Patient Population

All female patients (age ≥ 18 years) operated on BCBM between January 2008 and December 2019 in a single institution were eligible for this study. The cases with unknown outcome within 6 months after surgery were excluded from this study.

The indication on the microsurgical resection of BCBM was based on the interdisciplinary decision within the institutional tumor board. Common selection criteria for brain metastases resection were: the lesion size (>3 cm), presence of clinical symptoms, considerable peri-focal edema, non-eloquent location, as well as favorable KPS (>70%). In selected cases, patients with multiple brain metastases were also allocated for surgery of a singular brain metastases location for the treatment of the brain metastases-related mass-effect and/or diagnostic approval.

### 2.2. Data Management, Statistical Analysis, and Score Construction

The goal of the present study was the identification of independent predictors of poor (SS) and good (LS) outcome after BCBM microsurgery with subsequent construction of the appropriate risk scores for the estimation of outcomes at both timepoints. In accordance with the previously reported ranges of median postoperative overall survival for BCBM patients [42,43,44], SS was defined as a survival < 6 months, whereat LS was defined as a survival ≥ 3 years.

The following patients’ characteristics were recorded: age (at breast cancer and brain metastases diagnosis), the type of breast cancer surgery (mastectomy vs. breast-preserving surgery), time interval between the diagnosis of breast cancer and brain metastases, preoperative KPS scale at brain metastases diagnosis, occurrence of preoperative seizures, number (singular vs. multiple) and the receptor status of brain metastases, the original, modified and updated breast-GPA score values, pre-existing conditions (arterial hypertension, diabetes mellitus), and certain laboratory parameters at admission to assess the presence of anemia (hemoglobin), and inflammatory status (white blood cells and c-reactive protein).

At the neuropathology department, the receptor status of brain metastases was analyzed and estrogen receptor, progesterone receptor, and HER2 positive status were defined by immunoreactivity. The immunohistochemistry was used for defining the positive HER2 status as HER2 3+ (DAKO score) or HER2+ with HER2 gene amplification detected by fluorescence in situ hybridization. The nuclear staining of tumor response of tumor cells indicated the immunohistochemical estrogen receptor and progesterone receptor stains. The results were reported in percentage of positive tumor cell nuclei, with <1% nuclear positive reaction as receptor status negative and more than 1% of positive tumor cell nuclei as receptor status positive.

Data were analyzed using SPSS (version 27, SPSS Inc., IBM, Chicago, IL, USA) statistical software. The variables were reported in median values and interquartile ranges (IQR) between 25% and 75%, or as number of cases (with percentage), as appropriate.

First, the associations between the patients’ characteristics and SS/LS were evaluated in univariate analysis. After the dichotomization of the continuous variables based on the receiver operating characteristic (ROC) curves, the Chi-square (χ2 test) or Fisher exact tests between the potential predictors and the study endpoints were performed. The parameters with a *p* value of ≤ 0.10 were transferred to multivariate binary logistic regression analysis. Independent predictors of SS/LS which reached the level of significance (*p* ≤ 0.05) were included as the components of the SS and LS scores, respectively (as two separate scores). The score value for each component was calculated using the quotient of the appropriate adjusted odds ratios [45] value to the smallest aOR value of the significant parameters. The total score points were then calculated for each patient and each score. After the inclusion of the original, modified, and updated breast-GPA scores as the reference scores, the diagnostic accuracy of the new scores for the prediction of SS and LS was assessed using the ROC curves and comparison of the resulting areas under the curve [28]. Moreover, the clinical utility of the new risk scores for SS/LS were also evaluated for the prediction of overall survival by calculating the total (cumulative score value for each patient in the following manner:

“Total-Score” = “LS-Score” − “SS-Score”

Thereafter, Kaplan–Meier survival plots and log-rank test were performed to analyze the association between the Total-score and overall survival.

## 3. Results

### 3.1. Patients’ Characteristics

Between January 2008 and December 2019, 95 female patients with BCBM were included in the final analysis. The median overall survival was 16.0 months (IQR: 7.0–34.0). The median age at breast cancer diagnosis was 53.5 years (IQR: 45.8–63.3). At the time of brain metastases diagnosis, the median age was 60.0 years (IQR: 51.0–69.0). The distribution of breast cancer surgery type was equal (breast-preserving surgery: *n* = 51). The majority of the cases presented with singular and supratentorial brain metastases. A higher rate of negative HER2 status of brain metastases was identified. The clinical, histopathological and demographic characteristics are presented in Table 1 and Supplementary Table S1.

### 3.2. Parameters Related to Short Survival in Univariate Analysis

After BCBM surgery, 18 (18.9%) patients experienced short survival. Older individuals aged ≥ 65 years at breast cancer diagnosis were more prone to short survival (*p* = 0.054). Significant associations with short survival showed pre-existing comorbidities like arterial hypertension (*p* = 0.032) and diabetes mellitus (*p* = 0.021), as well as breast-preserving surgery (*p* = 0.034). In addition, short survival was more common in individuals with multiple brain metastases (*p* = 0.098). The remaining parameters showed no associations eligible for further assessment (see Supplementary Tables S2–S4).

### 3.3. Parameters Related to Long Survival in Univariate Analysis

Long survival was documented in 22 patients (23.2%) in our cohort. Positive HER2 status in brain metastases was significantly associated with good outcome (*p* = 0.045). Moreover, the patients’ age at breast cancer diagnosis (with the cutoff at 65 years, *p* = 0.035), the time interval between breast cancer and brain metastases diagnosis (≥3 years, *p* = 0.05), and preoperative KPS scale (≥90%, *p* = 0.025) showed significant associations with long survival. The results of the remaining univariate analysis for long survival predictors are presented in the Supplementary Tables S2–S4.

### 3.4. Multivariate Analysis for Short Survival and the SS-Score Construction

**Figure 1:**
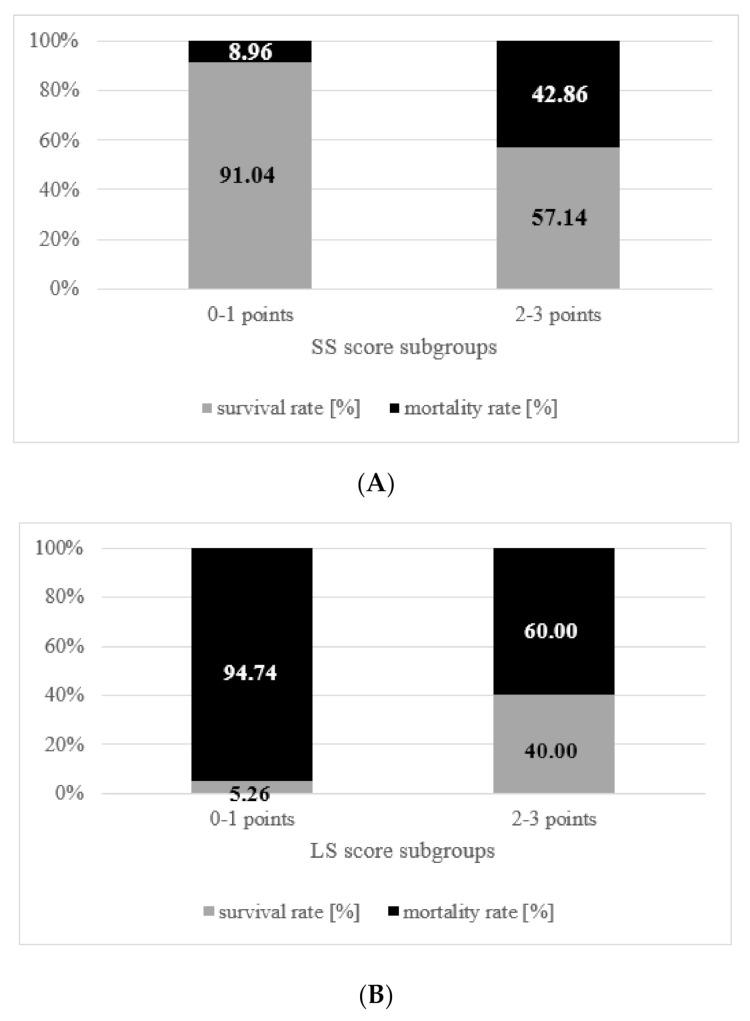
Bar charts showing the association between the SS- (**A**)/LS- (**B**) scores and the survival rates at appropriate timepoints (6 months and 3 years for the SS- and LS-scores, respectively). The outcomes were reported for each score after the dichotomization into low (0–1 points) and high (2–3 points) score values.

**Figure 2:**
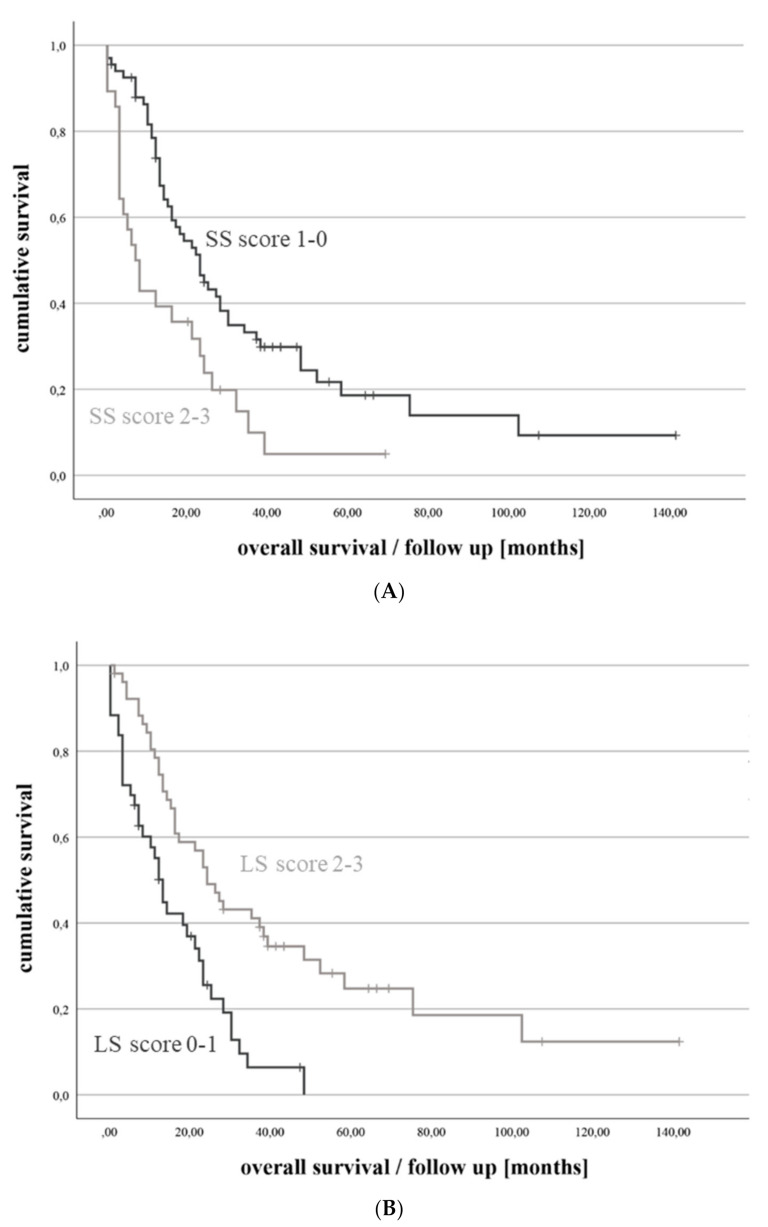
Kaplan–Meier curves demonstrate the survival differences in BCBM patients with low (0-1 points) and high (2–3 points) score value: (**A**) SS-scores (log rank test *p* = 0.002). (**B**) LS-scores (log rank test *p* < 0.001).

In the final multivariate logistic regression analysis (Table 2), we identified breast-preserving surgery (aOR 6.20, 95% CI 1.44–26.77, *p* = 0.015), multiple brain metastases (aOR 4.56, 95% CI 1.26–16.58, *p* = 0.021) and the age ≥ 65 years at breast cancer diagnosis (aOR 4.35, 95% CI 1.04–18.21, *p* = 0.044) as independent risk factors for short survival. Each of these parameters was valued with 1 point and included in the SS-score (range: 0 to 3 points). The summary SS-score value was calculated for every patient in the cohort. The distribution of short survival rates depending on the SS-score value is presented in Figure 1A. The Kaplan–Meier curve (see Figure 2A) also demonstrates the survival differences in the cohort according to the SS-score values.

### 3.5. Multivariate Analysis for Long Survival and the LS-Score Construction

The following three parameters showed significant associations with long survival in the multivariate analysis (Table 2): positive HER2 status in brain metastases (aOR 5.27, 95% CI 1.50–18.55, *p* = 0.010), time interval ≥ 3 years between breast cancer and brain metastases diagnosis (aOR 6.13, 95% CI 1.53–24.54, *p* = 0.010) and preoperative KPS ≥ 90% (aOR 4.36, 95% CI 1.12–16.92, *p* = 0.034). Accordingly, these independent long survival predictors were included in the LS-score. Each score component was weighted with 1 point and the summary LS-score value (range: 0 to 3 points) for each patient was thereafter calculated. The distribution of long survival rates throughout the LS-score is presented in Figure 1B. The Kaplan–Meier curve (see Figure 2B) shows the association between the LS-score and overall survival.

### 3.6. Diagnostic Accuracy of the SS-, LS-, and the GPA-Based Scores

**Figure 3:**
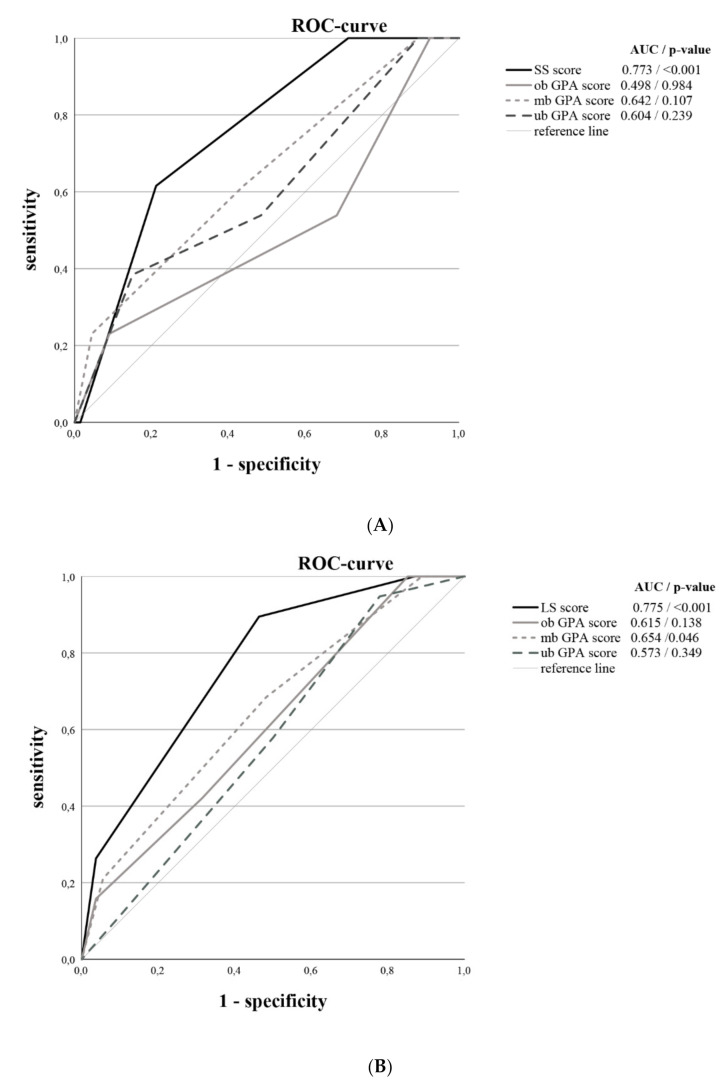
The receiver operating characteristic curves (ROC) demonstrate the diagnostic accuracy of different scores with regard to the prediction of short survival (**A**) and long survival (**B**) in BCBM patients. (**A**): demonstrated the SS-score and different GPA scores (inversed) for short survival (6 months survival). (**B**): demonstrated the LS-score and different GPA scores for long survival (3 years survival). Abbreviations: LS: long survival, SS: short survival, GPA: Graded Prognostic Assessment, ob: original breast, mb: modified breast, ub: updated breast, AUC: areas under the curve.

After the calculation of the original, modified and updated breast-GPA scores in our cohort as reference scores, the diagnostic accuracy of all scores were tested with regard to the prediction of short survival and long survival using the ROC analysis. As result, the SS-score (AUC: 0.773) was superior to the original (AUC: 0.498), modified (AUC: 0.642), and updated breast-GPA (AUC: 0.604) scores for short survival prediction. In turn, the LS-score (AUC: 0.775) showed higher diagnostic accuracy for long survival prediction, as compared to the original (AUC: 0.615), modified (AUC: 0.654), and updated breast-GPA (AUC: 0.573) scores (see Figure 3).

### 3.7. Total-Score

**Figure 4:**
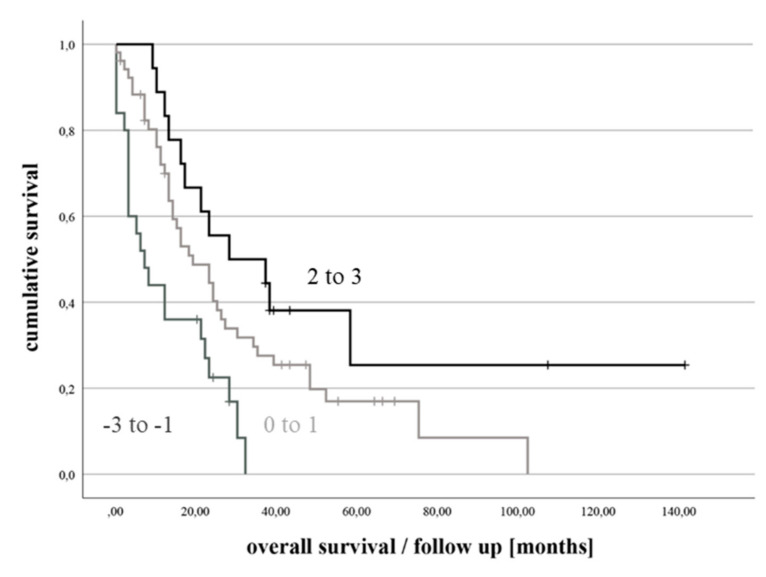
Kaplan-Meier curve for the association between the Total-score and overall survival (log-rank test *p* = 0.001). According to the Total-score value, the survival curves were divided into three subgroups: “−3 to −1 points”. “0 to 1 point”. “2 to 3 points”.

Upon the difference between the LS- and SS-scores, the Total-score (range: −3 to +3 points) values were also calculated (See Supplementary Figure S1). There was significant association between the Total-score and overall survival as shown in the Kaplan–Meier survival plot (See Figure 4).

## 4. Discussion

In virtue of changing demographic pattern towards higher rate of older females and improved healthcare in industrial countries, the optimization of treatment strategies for BCBM patients is of eminent importance. In this retrospective study, we identified independent predictors for short survival and long survival after BCBM surgery and constructed appropriate risk scores addressing each survival timepoint separately.

### 4.1. Survival Prediction after Brain Metastases Surgery: From Single Risk Factors to Risk Scores

Different patient and tumor characteristics were addressed as potential survival predictors in individuals with brain metastases. In particular, the patients’ age, KPS, comorbidities, primary tumor, and presence of extra-cranial metastases were frequently reported as prognostic factors influencing the postoperative survival [46,47,48,49].

The biological features of the primary tumor is the main determinant of overall survival after brain metastases surgery. As compared to other cancer types, breast cancer is characterized with slower growth, higher radio-sensitivity, and better prognosis [4,50]. However, depending on the individual risk profile, BCBM patients might also face a poor postoperative survival consisting of few months. Different clinical and demographic characteristics, such as age, number of brain metastases, presence of extra-cranial metastases, the histological subtype of breast cancer, as well as the KPS scale were previously reported as outcome-relevant parameters for breast cancer patients [16,17,26,30,31,32,33,34,35,51,52].

The implementation of the risk scores in the clinical practice allows the estimation of the cumulative effect of all significant predictors for the prognosticated outcome. In cancer healthcare, different clinical scores were constructed to predict the post-treatment survival. A recursive partitioning analysis [44] is probably the most commonly used early prognostication tool for breast cancer patients (the breast-RPA), but it also has several strong limitations and low clinical impact [46,53]. The breast-GPA score includes the KPS score, genetic subtype of breast cancer and age [26,38]. Number of brain metastases and changed age cutoff were added in the modified breast-GPA score by Subbiah et al. [37]. These two GPA score constructions represented a more accurate and precise score system for BCBM than the breast-RPA [26,37,40,53,54]. In 2020, Sperduto et al. presented an updated version of the breast-GPA which added the presence of extracranial metastases and the number of brain metastases [41]. Another study also identified extracranial metastases and the control of primary tumor as independent factors predicting overall survival [55].

As the above-mentioned scores were constructed on the general BCBM cohorts not limited to the surgical cases, the eligibility of these scores for BCBM patients selected for surgery remains unclear. In 2019, the modified breast-GPA showed higher diagnostic accuracy than the original GPA in a cohort of 169 patients, but only 54% of BCBM cases were operated and the AUC was very low (0.286) [40]. Riecke et al. addressed all three GPA scores and identified strong limitations of the GPA scores for long- and short-term survival [56]. Another recent study showed that the GPA score was not robust enough for outcome prediction in operated brain metastases patients regardless the cancer type [57]. Finally, the present study also verified a poor diagnostic accuracy of all three GPA-based scores for the prediction of short survival and long survival.

However, none of these scores were focused on the BCBM patients and their clinical value for the prediction of postoperative survival remains uncertain. The presented three negative (SS-Score) and three positive (LS-Score) predictors showed novel scores for the prediction of short survival and long survival in operated BCBM. These scores showed better prognostic accuracy, as compared to the original, modified, and updated breast-GPA scores. In turn, the cumulative score (the Total-score) based on the LS- and SS-scores showed strong association with overall survival throughout the whole postoperative follow-up time.

The survival predictors identified in this study are widely acknowledged prognostic factors both specifically for breast cancer patients (such as HER2 status [11,15,17,25], and time interval between breast cancer and brain metastases [58,59,60]), and for brain metastases patients regardless the cancer type: patients’ age [26,27,36,38], number of brain metastases [37,41,58,61,62], and preoperative KPS [26,38,47].

In our study, we identified breast-preserving surgery as independent predictor of poor survival after brain metastases surgery. This finding is of particular interest, since the breast-preserving surgery was not associated with worse prognosis and higher recurrence rate after primary breast cancer treatment in the majority of studies [9,63,64,65,66,67]. Fittingly, overall survival after breast-preserving surgery and mastectomy was comparable also in our cohort (see Supplementary Table S5). At the same time, breast-preserving surgery showed strong association with the risk of short survival after BCBM surgery independently of other outcome-relevant breast cancer and brain metastases-related features (such as the molecular status of breast cancer and the initial adjuvant treatment [9,10,11,17,68,69]).

The link between breast-preserving surgery and short survival after BCBM surgery might be related to eventual impact of residual tumor cells after breast-preserving surgery. This tumor cell burden might persist despite adjuvant treatment after breast-preserving surgery [70,71], and later sprout into the vascular and lymph nodal system. [72,73,74] It has been reported that the residual tumor cells are prone to metastasize [75], and their presence could be associated with worser outcome [74]. Two important aspects possibly contributing to the aggressiveness of breast cancer after breast-preserving surgery are the pan-resistance after initial therapy and the multi-drug resistance [76,77]. Our cohort treated with breast-preserving surgery demonstrated a higher rate of receptor conversion of HER2 in brain metastases which was reported to be associated with a poorer outcome after brain metastases diagnosis [78,79,80]. In addition, the rate of adjuvant radiotherapy after breast cancer was higher in individuals with breast-preserving surgery (Supplementary Table S5). These findings support the hypothesis of molecular changes under the adjuvant systemic and radiation therapy of the primary breast cancer [81], and the residual tumor cells might be the mediators of these molecular changes. Of note, the accurate pathways for distant metastases, especially for brain metastases after invasive breast cancer treatment, and particularly the exact role of residual tumor cells in the aggressiveness of BCBM is unknown [82].

We also analyzed the impact of neoadjuvant and adjuvant therapies of initial breast cancer on the prognosis of BCBM patients. So, (neo-)adjuvant treatment and the treatment response after initial breast cancer therapy showed no significant influence on the survival after BM surgery (Supplementary Tables S3 and S5). Moreover, the initial TNM stage and the Ki67 index of BC, as well as the presence of metastases before BM diagnosis also showed no association with the study endpoints.

During the construction of the novel scores for BCBM patients, we selected two opposing survival timepoints—a poor postoperative survival less than 6 months (SS) and a good outcome defined as survival longer than 3 years (LS). These time intervals reflect the common experience with prognosis after BCBM surgery reported in previous studies [42,43,44]. The selection of dual score goals enables the use of our prognostic tools separately for each survival timepoint. This approach might be reasonable during the selection of proper treatment strategy after BCBM diagnosis and subsequent consultation of patients. In particular, BCBM individuals with a high SS-score (and, accordingly, low or negative Total-score) might be considered for non-surgical management, especially at the presence of additional risk factors, which were not included in the scores. Additionally, vice versa, BCBM individuals with high LS-score (and positive Total-score) might be more eligible for surgical strategy.

Of note, the SS-, LS-, and Total-scores (just like any other prognostic scores) cannot present the sole basis for the decision on the advisability of surgical treatment. There are many additional essential arguments, such as the need for histological re-evaluation, presence of oncological treatment alternatives, anesthesiologic/cardiac risk profiles, and the patients’ willingness which must be incorporated in the final decision on the treatment strategy after the diagnosis of BCBM. Therefore, only an interdisciplinary consensus within the tumor boards warrants the selection of optimal treatment strategy in each individual case. Additionally, the presented prognostic scores are only additional tools which might be helpful in oncologic management of individuals with BCBM.

### 4.2. Limitation

The monocentric retrospective design, the sample size and incompleteness of the patient and follow-up data are the major limitations of this study. Accordingly, long survival could not be calculated for all patients in the final cohort. Although the BCBM patients in the cohort were selected for surgery upon the decision of the tumor board, but the selection criteria for BCBM surgery may differ between the neuro-oncologic centers, particularly when comparing the treatment strategies in different countries. In addition, adjuvant treatment of BCBM was not included in our analysis. This circumstance limits the generalizability of the results of the present study. Therefore, for successful implementation of the presented scores in the clinical practice, there is need for an internal as well as an external (prospective) validation of the scores. However, the next step of our study is the internal validation for underline the importance of our scores especially for operated BCBM patients. Moreover, we aim for implementable scores in the clinical practice. Therefore, we need an external validation which could be realized as a follow-on study in the future.

## 5. Conclusions

Age at breast cancer diagnosis (≥65 years), breast-preserving surgery as primary breast cancer therapy and multiple brain metastases are the essential risk factors for short survival after BCBM surgery. In turn, time interval between breast cancer and brain metastases diagnosis (≥3 years), preoperative KPS (≥90%) and the positive HER2 status in brain metastases are the major determinants of long survival. Based on the above-mentioned independent predictors, the new developed scores showed better prognostic accuracy than the previous score systems. In case of successful external validation, the presented SS-, LS-, and Total-scores could support the interdisciplinary decision for the selection of treatment strategy in individuals with BCBM.

## Figures and Tables

**Table 1:** Baseline characteristics of BCBM patients.

Parameter	Median (IQR) or Nr. (%)
Number of patients	95 (100%)
OS [months]	16.0 (7.0–34.0)
Preoperative KPS ≥90%	59 (62.1%)
Age at BC diagnosis [years]	53.5 (45.8–63.3)
Age at BM diagnosis [years]	60.0 (51.0–69.0)
Interval BC to BM [months]	44.5 (21.8–100.0)
Surgical treatment of BC	
mastectomy	44 (46.3%)
breast-preserving surgery (BPS)	51 (53.7%)
Histopathology of BC	
Invasive ductal	54 (56.8%)
Invasive lobular	10 (10.5%)
BC HER2 status	
positive	31 (32.6%)
negative	50 (52.6%)
BC ER status	
positive	47 (49.5%)
negative	34 (35.8%)
BC PR status	
positive	41 (43.2%)
negative	40 (42.1%)
Number of BM	
singular	64 (67.4%)
multiple	31 (32.6%)
BM location	
supratentorial	60 (63.2%)
infratentorial	35 (36.8%)
BM HER2 status	
positive	37 (38.9%)
negative	58 (61.1%)
BM ER status	
positive	48 (50.5%)
negative	47 (49.5%)
BM PR status	
positive	20 (21.1%)
negative	75 (78.9%)
Extracranial metastases	37 (38.9%)
Arterial hypertension	40 (42.1%)
DM	4 (4.2%)
Statin for hypercholesterinemia	12 (12.6%)
Neuroleptics	12 (12.6%)
Preoperative seizure	2 (2.1%)

Abbreviations: Nr.: number of cases, BC: breast cancer, BM: brain metastasis, IQR: interquartile ranges 25–75%, OS: overall survival, HER2: human epidermal growth factor receptor 2, ER: estrogen receptor, PR: progesterone receptor, DM: diabetes mellitus.

**Table 2:** Multivariate analysis for independent predictors of short (SS) and long survival (LS) after BCBM surgery.

Parameter	aOR	95% CI	*p*-Value	Score Value
**SS**				
Breast-preserving surgery	6.20	1.44–26.77	0.015	1
Arterial hypertension	2.84	0.83–9.70	0.095	_
Number of BM (>1)	4.56	1.26–16.58	0.021	1
Age at BC diagnosis (≥65 years)	4.35	1.04–18.21	0.044	1
Diabetes mellitus	12.48	0.77–201.22	0.075	_
**LS**				
HER2 RS in BM (positive)	5.27	1.50–18.55	0.010	1
Time interval BC—BM (≥3 years)	6.13	1.53–24.54	0.010	1
Preoperative KPS score (≥90%)	4.36	1.12–16.92	0.034	1
Age at BC diagnosis (< 65 years)	5.18	0.56–47.88	0.147	_

Abbreviations: aOR: adjusted odds ratio, CI: confidence interval, RS: receptor status, RC: receptor conversion, BM: brain metastases, HER2: human epidermal growth factor receptor 2, KPS: Karnofsky Performance Status scale.

## Data Availability

The data presented in this study are available on request from the corresponding author. The data are not publicly available due to ethical restrictions.

## Supplementary Materials

The following supporting information can be downloaded at: https://www.mdpi.com/article/10.3390/cancers14061437/s1, Figure S1: A score chart for the calculation of the SS-, LS- and Total-scores; Table S1: Overview of the receptor status combination, Ki67 index and TNM stage in primary breast cancer at diagnosis and G stage after surgical treatment or biopsy; Table S2: Univariate analysis (chi-square test) for the association between the baseline parameters and short (SS) and long survival (LS) after BCBM surgery. Table S3: Univariate analysis (chi-square test) for the association between the BC adjuvant and neoadjuvant treatment and short (SS) and long survival (LS) after BCBM surgery. Table S4: Univariate analysis (chi-square test) for the association between the BC tumor characteristics (TNM stage, KI67 index and metastases before BM diagnosis) and short (SS) and long survival (LS) after BCBM surgery. Table S5: Univariate analysis (chi-square test or Mann-Whitney test for continuous variables) for the association between the baseline parameters and BC surgical treatments (OR analysis for BPS as initially BC treatment).

## Abbreviations

aOR	Adjusted odds ratios
AUC	Areas under the curve
BCBM	Breast cancer brain metastases
CI	Confidence interval
GPA	Graded Prognostic Assessment
HER2	Human epidermal growth factor receptor 2
IQR	Interquartile ranges
KPS	Karnofsky Performance Status
LS	Long survival
ROC	Receiver operating characteristic
SS	Short survival
