# Supplementary material for: Prediction of Short and Long Survival after Surgery for Breast Cancer Brain Metastases
Source: Cancers (Basel). 2022 Mar 10;14(6):1437. doi: 10.3390/cancers14061437 (PMC8946189; doi:10.3390/cancers14061437)
Supplement: Supplementary file 1 [file cancers-14-01437-s001.zip › cancers-1602012-supplementary.pdf]

Supplementary figure S1: A score chart for the calculation of the SS-, LS- and Total-scores.

**SS score**			
Age at BC diagnosis	<65 years	0	
	**≥65 years**		1
BC surgical treatment	mastectomy	0	
	**Breast-preserving surgery**		1
Number of BM	1	0	
	**>1**		1
			SS

**LS score**			
BM HER2 RS	negative		0
	**positive**	1	
Preoperative KPS score	<90%		0
	**≥90%**	1	
Time interval BC-BM	<3 years		0
	**≥3 years**	1	
		LS	

**Total score**	LS - SS

Supplementary table S1: Overview of the receptor status combination, Ki67 index and TNM stage in primary breast cancer at diagnosis and G stage after surgical treatment or biopsy

**Parameter**		**Nr. (%)**
**Ki67 index**	≤20%	5 (5.3%)
	>20%	26 (27.4%)
	n.a.	64 (67.4%)
**ER/PR/HER2 status**	ER-PR-HER2-	19 (19.6%)
	ER+PR+HER2+	10 (10.3%)
	ER+PR+HER2-	25 (25.8%)
	ER+PR-HER2-	2 (2.1%)
	ER+PR-HER2+	10 (10.3%)
	ER-PR+HER2+	3 (3.1%)
	ER-PR+HER2-	3 (3.1%)
	ER-PR-HER2+	9 (9.3%)
	n.a.	16 (16.5%)
**Breast GPA tumor subtype**	Basal	19 (20.0%)
	Luminal A	30 (31.6%)
	Luminal B	10 (10.5%)
	HER2	21 (22.1%)
	n.a.	15 (15.8%)
**TNM-classification (at first diagnosis)**		
**T**	T0	1 (1.1%)
	T1	24 (25.3%)
	T2	18 (18.9%)
	T3	13 (13.7%)
	T4	6 (6.3%)
	n.a.	33 (34.7%)
**N**	N0	30 (31.6%)
	N1	10 (10.5%)
	N2	4 (4.2%)
	N3	2 (2.1%)
	n.a.	46 (48.4%)
**M**	M0	86 (90.5%)
	M1	8 (8.4%)
	n.a.	1 (1.1%)
**G**	G1	2 (2.1%)
	G2	19 (20.0%)
	G3	31 (32.6%)
	n.a.	43 (45.3%)
**metastases before BM diagnosis**	Yes	18 (18.9%)
	No	75 (78.9%)
	n.a.	2 (2.1%)

Abbreviations: +: positive, -: negative, OR: odds ratio, CI: confidence interval, Nr.: number, n.a.: not available, BM: brain metastases, T: tumor, N: node, M: metastasis, G: grade, ER: estrogen receptor, PR: progesterone receptor, HR: hormone receptor, HER2: human epidermal receptor 2, Ki67: marker of proliferation, GPA: Graded Prognostic Assessment; Information about GPA tumor subtype classification: basal=HR/HER2-negative.,Luminal B= HR/HER2-positive, Luminal A= HR-positive, HER2-negative, HER2= HR-negative/ HER2-positive

Supplementary table S2: Univariate analysis (chi-square test) for the association between the baseline parameters and short (SS) and long survival (LS) after BCBM surgery

	SS (poor prognosis)			LS (good prognosis)		
**Parameter**	**p-value**	**OR**	**95% CI**	**p-value**	**OR**	**95% CI**
**Tumor characteristics**						
Breast-preserving surgery	0.034	3.78	1.14-12.53	0.625	0.74	0.28-1.96
Time interval BC-BM (≥3 years)	0.793	0.76	0.27-2.18	0.050	3.01	0.99-9.12
Extracranial metastases	0.421	1.71	0.61-4.83	1.000	0.96	0.36-2.58
Number of BM>1	0.098	2.50	0.88-7.13	0.185	0.39	0.12-1.28
BM location (infratentorial)	0.278	1.96	0.70-5.54	1.000	0.94	0.34-2.55
HER2 RS in BM (positive)	0.178	0.38	0.12-1.26	0.045	2.89	1.07-7.79
ER RS in BM (positive)	0.609	0.74	0.26-2.08	0.806	1.20	0.58-2.49
PR RS in BM (positive)	0.108	0.18	0.02-1.44	0.139	2.94	0.75-11.52
**Patients' characteristics**						
Age at BC diagnosis (≥65 years)	0.054	3.13	1.02-9.60	0.035	0.14	0.02-1.10
Age at BM diagnosis (≥65 years)	0.425	1.66	0.59-4.65	0.210	0.45	0.16-1.29
Preop. KPS score (≥90 %)	0.285	0.54	0.19-1.52	0.025	3.75	1.14-12.29
Preop. anemia (<12g/dl)	0.292	2.24	0.66-7.61	0.741	1.25	0.34-4.54
Preop. WBC (>10/nl)	1.000	1.06	0.37-3.05	0.437	0.58	0.20-1.64
Preop. CRP (>0.5mg/dl)	0.515	1.64	0.50-5.42	0.749	0.62	0.16-2.43
Arterial hypertension	0.032	3.50	1.18-10.35	1.000	0.88	0.33-2.35
Diabetes mellitus	0.021	15.20	1.48-156.21	0.570	0.74	0.65-0.84
Statin for hypercholesterinemia	1.000	0.89	0.18-4.51	1.000	1.13	0.27-4.68
Neuroleptics	1.000	0.89	0.17-4.51	0.722	0.55	0.11-2.73

Abbreviations: BM: brain metastases, KPS: Karnofsky Performance Status scale, BC: breast cancer, HER2: human epidermal growth factor receptor 2, ER: estrogen receptor, PR: progesterone receptor OR: odds ratio, CI: confidence interval, SS: short survival = poor prognosis/ survival <6 months, LS: long survival = good prognosis / survival ≥3 years, preop.: preoperative, postop.: postoperative, WBC: white blood cells, CRP: C-reactive protein

Supplementary table S3: Univariate analysis (chi-square test) for the association between the BC adjuvant and neoadjuvant treatment and short (SS) and long survival (LS) after BCBM surgery

	**SS (poor prognosis)**			**LS (good prognosis)**		
**Parameter**	**p-value**	**OR**	**95% CI**	**p-value**	**OR**	**95% CI**
**<u>Neoadjuvant treatment</u>**						
Paclitaxel	0.483	_	_	0.643	1.83	0.28-12.07
Docetaxel	1.000	2.00	0.11-36.31	1.000	0.71	0.10-5.04
Pertuzumab	1.000	_	_	1.000	1.50	0.11-20.30
Trastuzumab	0.492	_	_	0.643	1.83	0.28-12.07
Carboplatin	1.000	_	_	1.000	1.50	0.11-20.30
DC or EC	0.138	_	_	0.660	1.78	0.25-12.45
**<u>Adjuvant treatment</u>**						
Adjuvant radiotherapy	0.406	2.00	0.60-6.67	0.595	0.71	0.26-1.96
Adjuvant systemic treatment and chemotherapy	0.762	0.79	0.25-2.53	0.573	1.56	0.43-5.27
Adjuvant chemotherapy	1.000	0.99	0.35-2.78	0.622	1.37	0.51-3.71
Aromatase inhibitors	0.506	1.48	0.41-5.41	1.000	0.98	0.27-3.52
Tamoxifen	0.693	1.51	6.26	1.000	1.00	0.25-4.08
Antihormonal therapy	0.381	1.64	0.54-5.00	1.000	0.85	0.27-2.65
Chemotherapy	1.000	0.99	0.35-2.78	0.622	1.37	0.51-3.71
FEC	1.000	0.71	0.07-7.16	0.691	0.60	0.10-3.55
EC	0.157	5.00	0.79-31.77	0.477	0.49	0.11-2.21
Docetaxel	0.388	0.28	0.03-2.60	0.468	1.84	0.43-7.77
Paclitaxel	0.653	0.43	0.05-4.05	0.240	2.86	0.63-12.92
Pertuzumab	0.587	_	_	0.118	4.59	0.71-29.80
**<u>Response rate to (neo)adjuvant treatment (Nr.[%])</u>**						
Presence of residual disease (23 [24.2%])	0.765	1.22	0.38-3.88	0.785	0.813	0.26-2.55
Absence of residual disease (70 [73.7%])						
n.a. (2 [2.1%])						

Abbreviations: BC: breast cancer, OR: odds ratio, CI: confidence interval, SS: short survival = poor prognosis/ survival <6 months, LS: long survival = good prognosis / survival ≥3 years, DC= Doxorubicin/ Cyclophosphamide), EC= Epirubicin/Cyclophosphamide, FEC= Fluorouracil/ Epirubicin/ Cyclophosphamide), n.a.: not available, Nr.: number

Supplementary table S4: Univariate analysis (chi-square test) for the association between the BC tumor characteristics (TNM stage, KI67 index and metastases before BM diagnosis) and short (SS) and long survival (LS) after BCBM surgery

	**SS (poor prognosis)**			**LS (good prognosis)**		
**Parameter**	**p-value**	**OR**	**95% CI**	**p-value**	**OR**	**95% CI**
**BC tumor characteristics**						
Ki67 index >20%	0.613	1.81	0.25-13.21	0.350	0.35	0.05-2.51
T stage >1	0.744	0.774	0.21-2.88	0.567	0.67	0.21-2.16
N stage >0	0.374	1.95	0.60-6.39	0.202	0.49	0.17-1.37
M stage >0	0.341	0.51	0.16-1.62	0.387	1.80	0.57-5.68
G stage >1	0.498	2.57	0.15-44.00	1.000	0.85	0.05-14.39
metastases before BM diagnosis	0.399	1.64	0.56-4.79	1.000	1.04	0.37-2.92

Abbreviations: OR: odds ratio, CI: confidence interval, T: tumor, N: node, M: metastasis, G: grade, Ki67: marker of proliferation, BM: brain metastases

Supplementary table S5: Univariate analysis (chi-square test or Mann-Whitney test for continuous variables) for the association between the baseline parameters and BC surgical treatments (OR analysis for BPS as initially BC treatment).

	**mastectomy**	**BPS**	**p-value**	**OR**	**95%**
	Number (%)				
**BM characteristics**					
BM location (infratentorial)			0.524	0.72	0.31-1.67
infratentorial	18 (40.9%)	17 (33.3%)			
supratentorial	26 (59.1%)	34 (66.7%)			
Number of BM (multiple)			0.516	0.73	0.31-1.72
singular	28 (63.6%)	36 (70.6%)			
multiple	16 (36.4%)	15 (29.4%)			
HER2 receptor conversion (converted)			0.032	8.46	1.01-71.14
converted	1 (2.6%)	8 (18.6%)			
identic	37 (97.4%)	35 (81.4%)			
**Patients' characteristics**					
Age at BC diagnosis (≥65 years)			0.453	0.64	0.24-1.73
≥65 years	11 (25.0%)	9 (17.6%)			
<65 years	33 (75.0%)	42 (82.4%)			
Preop. KPS (<90%)			0.834	1.13	0.49-2.60
<90%	16 (36.4%)	20 (39.2%)			
≥90%	28 (63.6%)	31 (60.8%)			
Extracranial metastases			0.526	1.42	0.61-3.27
no	28 (65.1%)	29 (56.9%)			
yes	15 (34.9%)	22 (43.1%)			
Bone metastases			0.483	1.43	0.55-3.73
no	34 (79.1%)	37 (72.5%)			
yes	9 (20.9%)	14 (27.5%)			
Liver metastases			0.117	2.60	0.84-8.01
no	38 (88.4%)	38 (74.5%)			
yes	5 (11.6%)	13 (25.5%)			
Lung metastases			1.000	0.83	0.22-3.07
no	38 (88.4%)	46 (90.2%)			
yes	5 (11.6%)	5 (9.8%)			
**BC characteristics**					
BC histopathology (invasive ductal)			0.491	2.02	0.51-8.00
invasive lobular	6 (20.7%)	4 (11.4%)			
invasive ductal	23 (79.3%)	31 (88.6%)			
BC HER2 status (negative)			0.823	0.89	0.36-2.19
negative	24 (63.2%)	26 (60.5%)			
positive	14 (36.8%)	17 (39.5%)			
Adj. BC treatment: radiation			0.009	3.32	1.36-8.10
no	21 (47.7%)	11 (21.6%)			
yes	23 (52.3%)	40 (78.4%)			
Adj. BC treatment: antihormonal			1.000	1.03	0.41-2.60
no	33 (75.0%)	38 (74.5%)			
yes	11 (25.0%)	13 (25.5%)			

Adj. BC treatment: Trastuzumab			0.335	1.70	0.64-4.55
no	36 (81.8%)	37 (72.5%)			
yes	8 (18.2%)	14 (27.5%)			
Adj. BC treatment: chemotherapy			0.679	0.78	0.35-1.76
no	18 (40.9%)	24 (47.1%)			
yes	26 (59.1%)	27 (52.9%)			
Adj. BC treatment: antihormonal and/or chemotherapy			0.478	0.68	0.26-1.77
no	9 (20.5%)	14 (27.5%)			
yes	35 (79.5%)	37 (72.5%)			
	**mastectomy (IQR)**		**BPS (IQR)**		**p-value**
Median overall survival after BC [months]	63.0 (42.3-12.8)		58.0 (41.0-128.0)		0.923
Median overall survival after BM [months]	17.5 (11.0-29.5)		16.0 (3.0-37.0)		0.382
Median time interval BC-BM [months]	39.5 (21.3-112.8)		46.5 (23.3-99.3)		0.973

Abbreviations: BM: brain metastases, KPS: Karnofsky Performance Status scale, BC: breast cancer, HER2: human epidermal growth factor receptor 2, ER: estrogen receptor, PR: progesterone receptor OR: odds ratio, CI: confidence interval, preop.: preoperative, adj.: adjuvant, BPS: breast-preserving surgery, IQR: interquartile ranges (between 25% and 75%).
